# The role of human immunity and social behavior in shaping influenza evolution

**DOI:** 10.1371/journal.ppat.1006432

**Published:** 2017-08-03

**Authors:** Adam J. Kucharski, Marc Baguelin

**Affiliations:** 1 Centre for the Mathematical Modelling of Infectious Diseases, London School of Hygiene & Tropical Medicine, London, United Kingdom; 2 Respiratory Diseases Department, Public Health England, London, United Kingdom; University of Pittsburgh, UNITED STATES

**Fig 1:**
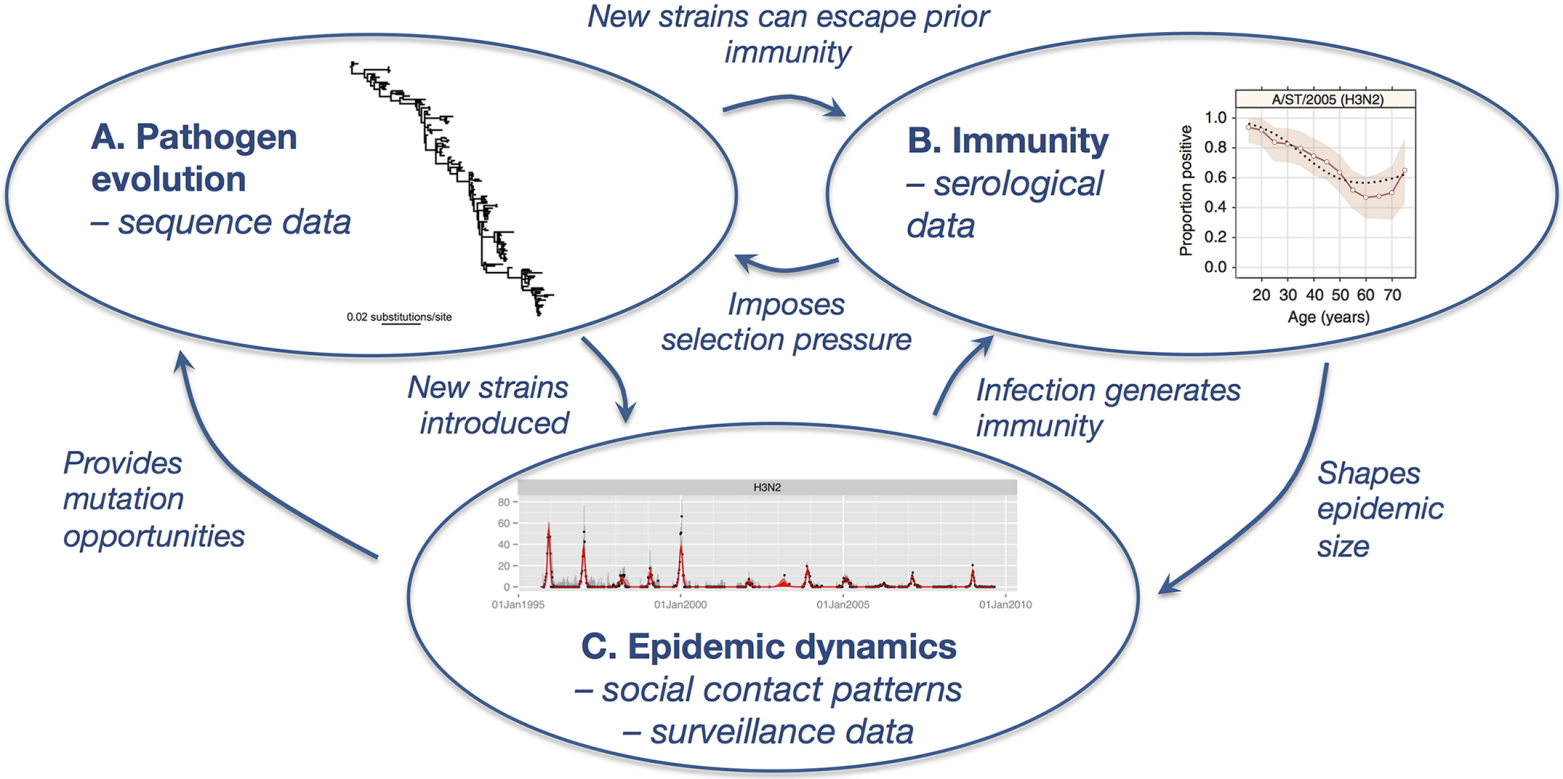
Influenza immunological, epidemiological, and evolutionary dynamics. Influenza undergoes antigenic evolution over time, escaping prior immunity and generating new epidemics. These epidemics can in turn give rise to new strains. To fully understand the dynamics of such infections, it is therefore important to consider the interactions between these 3 processes. Illustrative figures shown for influenza A/H3N2: (A) global phylogeny [25], (B) proportion of individuals with detectable titers in China [26], and (C) clinical reports in the United Kingdom [14].

Understanding the emergence of new influenza strains is crucial for seasonal vaccine strain selection and for assessing pandemic risk [1,2]. In particular, recent mismatches between vaccine composition strains and subsequent circulating viruses have led to low vaccine effectiveness and highlighted the need for improved predictions about which antigenic variants will be dominant during future epidemics [3]. During the process of selecting vaccine strains from candidate circulating strains, sequence data can provide an indirect measurement of viral transmission fitness by revealing which new genetic variants have successfully become fixed globally [2,4]. Moreover, recent work has shown the importance of age-specific mixing and travel patterns in shaping the global circulation of influenza strains [5]. Current influenza surveillance also includes analysis of epidemiological dynamics, with local outbreaks used as a proxy for transmissibility of viruses [2]. However, the relationship between influenza transmission dynamics, population herd immunity, and viral evolution remains poorly understood. Specifically, there is a need to understand how selection pressure imposed via population-level immunity and transmission combine to shape the observed evolution of influenza viruses (Fig 1).

In recent years, considerable resources have been committed to better monitor influenza evolution and resulting global diversity, most notably through the development of national influenza centers, with around 150 countries currently participating [6]. This effort has resulted in a wealth of data being accumulated, in particular, genetic sequences. During the same period, serology has increasingly been employed to measure individual immunity against specific influenza strains [7,8], and there have been calls for a “World Serology Bank” to curate such data [9]. Studies of social mixing patterns have also started to elucidate the role of age-specific social contacts in the transmission of respiratory infections [10–12].

Given this growth in data, there is now potential to develop a comprehensive framework to bring disparate data streams together to jointly analyze influenza evolution, immunity, and transmission. For example, how do antigenic drift, herd immunity, and human behavior combine to influence outbreaks? Although patterns of cross-reactive antibody responses against circulating influenza viruses have been well characterized [13] and the landscape of antibody responses can be mapped at the individual host level [7], it has been challenging to estimate the population-level transmission potential of a specific virus from available data. Previous infections may generate cross-protective responses against related influenza strains, which will in turn reduce the transmissibility of such strains within a population. However, there is currently a critical link missing between the measurement of individual serological responses and analytic tools to predict transmission dynamics.

Influenza transmission depends both on the extent of cross-protective immunity within a population—which is likely to vary between different age groups—and transmission-relevant contact patterns within and between those groups. For example, younger age groups typically have higher antibody titers against recent influenza strains compared to older individuals [8] but also have more social contacts [11]. Faced with a novel infection, children would be expected to contribute most to transmission, but this may not be the case if they had preexisting immunity to a homologous or related strain [14]. The overall transmission potential of an infection in a population can be measured using the effective reproduction number, R, defined as the average number of secondary cases generated by a typical infectious individual, which incorporates information about both population immunity and infectious contacts.

**Fig 2:**
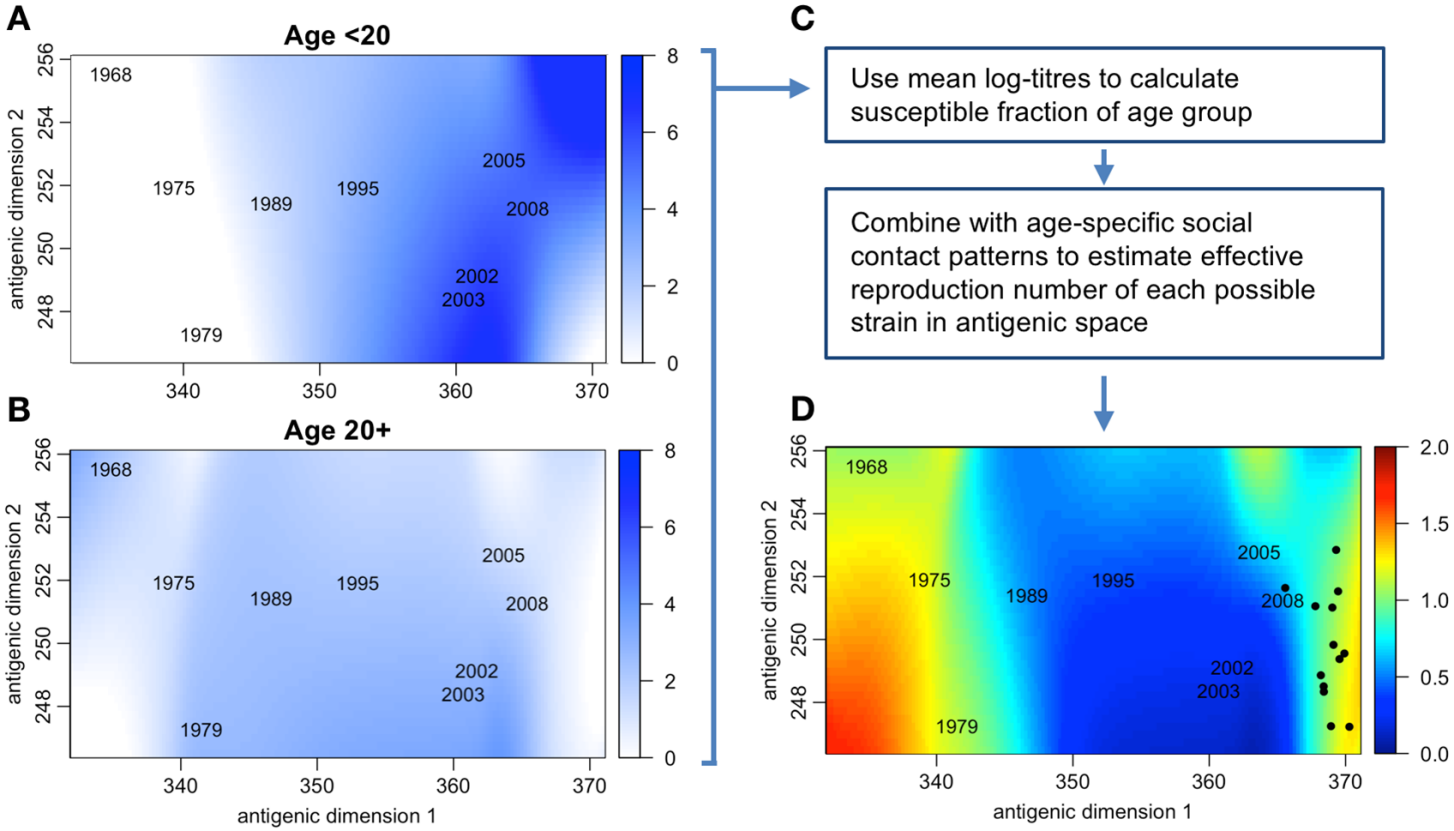
Using serology and social contacts to explore the evolutionary dynamics of influenza A/H3N2. (A, B) Mean log neutralization titer in age <20 and 20+ groups measured in 2009 against 9 strains in different antigenic locations, calculated using an “antibody landscape” smoothing technique [7]. Data from 151 individuals in Guangdong, China [8,26]; year labels show antigenic location of the 9 test strains used to estimate the landscape. (C) Log titers can be converted into the proportion of each group susceptible against each strain [15] and combining with age-stratified social-contact data [11] to calculate the effective reproduction number (defined as the average number of secondary cases generated by a typical infectious host) for each strain location. (D) Effective reproduction number for different strains in antigenic space. Black dots show locations of strains that circulated in the 2 years after serological samples were collected (i.e., 2009–2011) [7]. The basic reproduction number (i.e., the average number of secondary cases generated by a typical infectious host in a fully susceptible population) is assumed to be 2. Code and data are available at: https://github.com/adamkucharski/antigenic-evo.

The increasing availability of serological and social-contact data presents an opportunity to combine immunological and epidemiological dynamics to estimate the overall transmission potential of different strains. To illustrate how such an analysis could be implemented, we combined publicly available serological and social-contact data from southern China to estimate the R of different strains of influenza A/H3N2 in 2009. R also provides a threshold value that characterizes the ability of a particular infection to spread in a population with preexisting immunity: for R values above 1, strains have epidemic potential. In the China data, the under-20-years-old age group had high titers on average against recent isolates (Fig 2A), whereas the older age group had a different immunity profile, with lower average titers and most immunity against strains further in the past (Fig 2B). The values of R for strains at different points in the A/H3N2 antigenic space were calculated by converting the under- and over-20 immune profiles into measures of age-specific susceptibility [15], then combining these susceptibility estimates with the social mixing patterns of the 2 age groups. The resulting transmission fitness landscape indicated there were 2 regions of antigenic space that contain strains with high fitness, 1 near strains that circulated in the 1970s and 1 near the most recent isolates (Fig 2C).

In this example, post-2009 influenza isolates (shown as black dots) were generally located in the higher-fitness region predicted by the model. The correspondence between the fitness landscape and future isolates in this simple illustration indicates that the new emerging strains were correctly characterized as having a higher transmission potential. This approach could also quantify the risk posed by previously circulating strains: the 2009 influenza A/H1N1p virus, which was antigenically similar to pre-1957 strains, could spread because there was not sufficient humoral immunity in younger age groups [16,17]; there has also been concern about influenza A/H2N2, which has not circulated in humans since 1968 [18]. Similarly, the results in Fig 2 suggest that some pre-1979 A/H3N2 strains could have high transmissibility if reintroduced into a contemporary population. As well as identifying possible reemergence of homosubtypic pandemic viruses, the landscape of transmission fitness for seasonal influenza strains could also provide information about the potential emergence of novel influenza viruses, as there could be cross-protective responses against novel viruses within the same phylogenetic group [19,20].

A limitation of the example shown in Fig 2 is that the serological data are cross sectional and only capture population immunity when samples were taken in 2009. There is also evidence that specificity of immune responses can vary with age [21,22], with certain cohorts having monoclonal antibodies that target different antigenic sites to other groups; this may further shape the emergence and circulation of drifted viruses during an influenza season. The next step would be to link longitudinal serology with mathematical models of infection and antibody responses to explore how the landscape of population immunity accumulates over time. This would also make it possible to “rewind” history by removing recent periods of infection from the modelled immune landscapes and hence generate estimates for the historical fitness of specific influenza strains.

Such analysis could bring key scientific and public health benefits. First, it could lead to local risk mapping for novel influenza strains. Using serological and social-contact data in conjunction with mechanistic models, it would be possible to map herd immunity and transmission potential in different age groups and identify potential “blind spots” against specific strains [23]. This could help quantify how the background of population immunity influences the effectiveness of repeated vaccination in the context of existing national immunization campaigns. Second, the analysis could reveal which strains pose the greatest epidemic risk and hence which viruses vaccination may need to target. In future, this could lay the foundations for “personalized immunization” strategies [24], which would aim to increase vaccine effectiveness by employing different vaccine compositions depending on age and location. Third, by comparing estimates of historical influenza fitness landscapes to the observed trajectory of influenza antigenic evolution, it would be possible to establish what factors are most important in driving the emergence of new strains. In particular, there is potential to understand whether certain age groups and geographic locations contribute disproportionately to the selection pressure acting on circulating influenza viruses and hence what forms of surveillance would enable influenza evolution to be predicted more accurately in future.
